# The diagnostic value plasma procalcitonin (PCT) and serum amyloid A protein (SAA) in children with mycoplasma pneumonia

**DOI:** 10.5937/jomb0-53616

**Published:** 2025-06-13

**Authors:** Baoyue Wang, Yao Zheng, Linnan Jia

**Affiliations:** 1 Jilin Province FAW General Hospital, Department of Electrodiagnosis, Changchun, China

**Keywords:** procalcitonin, amyloid protein A, children, mycoplasma pneumonia, prokalcitonin, amiloidni protein A, deca, pneumonija izazvana mikoplazmom

## Abstract

**Background:**

To evaluate the diagnostic value of pulmonary ultrasound combined with plasma procalcitonin (PCT) and serum amyloid A protein (SAA) in children with Mycoplasma pneumoniae pneumonia (MPP).

**Methods:**

Clinical data of 80 children with community-acquired pneumonia who underwent lung ultrasound examinations and were admitted to our hospital from January 2023 to December 2023 were collected. Children with MPP were divided into an MPP group and a non-MPP group on the grounds of whether they were MPP patients. Meanwhile, PCT and SAA levels were checked, and lung ultrasound and PCT and SAA examination results were collected and compared. The ROC curve was used to evaluate the value difference of the three alone and in combination in diagnosing mycoplasma pneumonia.

## Abstract (sr)

**Uvod:**

Procena dijagnostičke vrednosti ultrazvuka pluća u kombinaciji sa plazma prokalcitoninom (PCT) i serumskim amiloidnim proteinom A (SAA) kod dece sa pneumonijom izazvanom Mycoplasma pneumoniae (MPP)

**Metode:**

Za analizu su prikupljeni klinički podaci osam - desetoro dece sa vanbolničkom pneumonijom koja su primljena u našu bolnicu od januara 2023. do decembra 2023. godine, a koja su prošla ultrazvučni pregled pluća. Deca sa MPP su podeljena na MPP grupu i ne-MPP grupu, u zavisnosti od toga da li su bila MPP pacijenti. Isto vreme, proveravani su nivoi PCT i SAA, a rezultati ultrazvuka pluća, kao i vrednosti PCT i SAA, su prikupljeni i upoređeni. ROC kriva je korišćena za procenu razlike u vrednosti pojedinačnog i kombinovanog korišćenja ova tri parametra u dijagnostikovanju pneumonije izazvane mikoplazmom.

**Rezultati:**

U poređenju sa decom koja nemaju MPP, kod dece sa MPP su primećene značajne razlike u više aspekata ultrazvučnog prikaza pluća, kao i u nivoima PCT i SAA. Logistička regresiona analiza je dodatno ukazala da su prisustvo povećanih/spojenih B-linija, povišen nivo PCT i povi{en nivo SAA povezani sa manjim rizikom od MPP, dok je mala količina pleuralnog izliva identifikovana kao faktor rizika za MPP. Logistički regresioni model zasnovan na ove četiri promenljive pokazao je dobru prediktivnu vrednost za MPP.

**Zaključak:**

Abnormalne karakteristike ultrazvuka pluća, kao što su povećane/spojene B-linije, mala količina pleuralnog izliva, kao i nivoi PCT i SAA, su povezane sa MPP kod dece. Prisustvo male količine pleuralnog izliva ukazuje na veći rizik od bolesti nego sama MPP. Merenje nivoa PCT i SAA, zajedno sa ultrazvučnim pregledom pluća, može da poboljša tačnost u razlikovanju ranog stadijuma pneumonije koja ja izazvana mikoplazmom od one koja to nije.

## Introduction

The incidence rate of Mycoplasma pneumoniae pneumonia (MPP) has gradually increased in recent years, with a risk of developing into refractory MPP [1]. The study of Álvaro et al. [2] in Europe showed that the incidence rate of mycoplasma pneumonia increased from 3.5% to 8.2%. A study by Wetzke et al. [3] in Germany showed that MPP accounted for 29.5% of hospitalised pediatric pneumonia cases and 42.5% of non-hospitalised pediatric pneumonia cases. According to the survey, during the MPP epidemic, the detection rate of MPP in children with pneumonia ranged from 21.5% to 81.8%, influenced by age [4]. MPP in children was dominant in community-acquired pneumonia [5]. This suggested that MPP in children is becoming one of the main pathogens leading to hospitalisation for pediatric pneumonia, and attention should be paid to its early diagnosis accuracy and timeliness. However, these epidemiological changes pose new challenges to clinical diagnosis and medication decision-making.

At present, the diagnosis of MPP mainly relies on pathogenic tests and serological antibody tests. Existing pathogenic tests [6] and serological antibody tests [7] pose certain difficulties in diagnosing MPP in children [8].

Scherrer et al. [9] showed that by relying solely on traditional pathogenic methods, such as culture and single PCR detection, the positive detection rate of mycoplasma pneumonia is lower. Cai et al. [10] showed that the clinical manifestations of pediatric pneumonia caused by different pathogens can be very similar, making diagnosis challenging. Wang et al. [11] also showed that the positive rate of sputum pathogen testing in children with pneumonia is relatively low, highlighting the need for more sensitive methods.

It can be seen that more sensitive pathogenic detection methods need to be developed, and more reliable auxiliary diagnostic indicators need to be sought. Meanwhile, Jourquin et al. [12] applied lung ultrasound to detect the progression of lesions in a calf mycoplasma pneumonia model, demonstrating the potential of ultrasound to observe the extent of lesions dynamically. Hu et al. [13] used deep learning technology to analyse the imaging features of chest films of children with pneumonia to identify the pathogens. The results showed certain differences in imaging manifestations between bacterial pneumonia and viral/mycoplasma pneumonia.

Research has shown that imaging studies have found that there are significantly more patients with pleural effusion, pulmonary complications, and bronchopneumonia imaging features in children with MPP than in those with pulmonary MPP [14]. Some studies have also found that measuring the length and area of pulmonary parenchymal lesions using lung ultrasound can evaluate the extent of MPP in children and provide a basis for clinical treatment plans [15]. The combination of chest X-ray and various inflammatory markers for differential diagnosis of MPP can help improve the current status of early diagnosis [16].

Liu et al. [17] showed that, compared to bacterial pneumonia, procalcitonin (PCT) levels in children with mycoplasma pneumonia were lower. Wrotek et al. [18] also found that, compared to bacterial pneumonia, children with viral pneumonia and mycoplasma pneumonia have lower levels of PCT. It can be seen that PCT is expected to become an important blood testing indicator for the clinical differentiation of MPP in children. Yan et al. [19] found that, compared with healthy children, the levels of Serum amyloid A protein (SAA) in children with MPP significantly increased, indicating that SAA can serve as an inflammatory marker for evaluating mycoplasma pneumonia and can be tested in conjunction with PCT. Research has found that SAA and PCT are specific markers for diagnosing early MP infection in children [20].

On the grounds of the limited research on the comprehensive application of lung ultrasound, PCT, and SAA in the diagnosis of mycoplasma pneumonia in children, this study assumes that lung ultrasound combined with blood PCT and SAA can effectively improve the clinical differentiation accuracy and diagnostic efficacy of MPP in children. Therefore, this study collected case data of children with community-acquired pneumonia and reviewed the results of relevant lung ultrasound examinations and blood PCT and SAA levels. The ROC curve was used to analyse the value of lung ultrasound combined with serum PCT and SAA in diagnosing mycoplasma pneumonia in children. This is to optimise the diagnostic process of pediatric MPP further, improve the sensitivity and specificity of diagnosis, and provide more clinically valuable data references for clinical diagnosis.

## Materials and methods

### Baseline information

This study collected clinical data from 80 children with community-acquired pneumonia who were admitted to Jilin Province FAW General Hospital in Changchun from January 2023 to December 2023 and underwent lung ultrasound examination. This study has been reviewed and approved by the Medical Ethics Committee of our hospital with approval code No.2022LC037, and the research design meets the ethical requirements of the Helsinki Declaration. This study employed a consecutive sampling method, where all eligible children with community-acquired pneumonia who underwent lung ultrasound examination during the study period were included in the sample. We recruited patients over 1 year, from January 2023 to December 2023. The sample size was calculated based on the estimated incidence of community-acquired pneumonia in children, which is approximately 15 episodes per 1000 children per year [21]. Assuming a desired power of 80% and a significance level of 0.05, a minimum sample size of 78 children was required to detect a significant difference in lung ultrasound findings between children with mild and severe pneumonia.

A total of 120 children with suspected community-acquired pneumonia were screened for eligibility between January 2023 and December 2023. The inclusion criteria were: (i) age 3–12 years old, (ii) clinical manifestations consistent with community-acquired pneumonia, including symptoms of respiratory infections such as fever, cough, and wheezing, (iii) chest imaging examination indicating signs of pneumonia, and (iv) informed consent from parents. Exclusion criteria included concomitant respiratory system diseases (n=15), severe organ dysfunction (n=8), malignant tumours or haematological diseases (n=5), and mental nervous system diseases or chest lesions not suitable for chest scanning (n=2). After applying the inclusion and exclusion criteria, 90 children were eligible for the study. Of these, 10 children declined to participate or did not complete the study, resulting in a final sample of 80 children. The 80 children were then divided into two groups based on whether they met the diagnostic criteria for Mycoplasma pneumoniae pneumonia (MPP): (1) MPP group, defined as a 4-fold increase in serum specific IgG antibodies in both acute and recovery phases, or serum specific IgM antibody detection (particle agglutination method, IgM>1:160), or Mycoplasma DNA or RNA (PCR) test positive (n=40); and (2) non-MPP group, defined as not meeting any of the above criteria (n=40).

### Research method

### Comparison of baseline data

Using a baseline data collection Table 1, two investigators collected and compared data on gender, age, clinical manifestations (fever, cough, wheezing), course of disease, and whether antibiotics were used before treatment.

**Table 1:** Comparison of Baseline Data and Pulmonary Ultrasound Examination Results between MPP and Non-MPP Groups of Pediatric Patients. Mycoplasma pneumoniae pneumonia (MPP)

Baseline Information	MPP Group<br>(n=26)	Non-MPP Group<br>(n=54)	χ^2^/t	P
** Gender (Male/Female) **	18/8	32/22	0.745	0.388
** Age (Year old) **	7.62±1.50	6.87±2.33	1.723	0.089
** Clinical manifestation **				
** ‣ Fever **	23	48	0.003	0.955
** ‣ Cough **	21	42	0.094	0.759
** ‣ Pant **	9	13	0.978	0.323
** Course of disease (d) **	7.19±3.12	6.65±3.23	0.214	0.644
** History of Antibiotic Application **	7	12	0.713	0.478
** Pulmonary Ultrasound **				
** Pulmonary parenchymal lesions **	20 (76.92)	32 (59.26)	2.407	0.121
** B line increase/fusion **	15 (57.69)	44 (81.48)	5.130	0.024
** Small amount of pleural effusion **	6 (23.08)	25 (46.30)	3.987	0.046
** Bronchograms **	5 (19.23)	23 (42.59)	4.210	0.040
** Pleural thickening **	5 (19.23)	8 (14.81)	0.251	0.616
** Lung Consolidation **				
** ‣ Bilateral lower lung area **	12	13	1.127	0.289
** ‣ Right middle lobe **	4	11	0.964	0.326
** ‣ Other parts **	** 4 **	** 8 **	** 0.165 **	** 0.685 **
** Consolidation diameter **				
** ‣ <1cm **	**14 **	** 18 **	** 0.964 **	** 0.326 **
** ‣ 1–3cm **	** 6 **	** 14 **		
** Number of consolidation **				
** ‣ 1–3 pieces **	** 12 **	** 21 **	** 0.165 **	** 0.685 **
** ‣ >3 pieces **	** 8 **	** 11 **		

### Lung ultrasound examination method

It designs recording charts, registers patient information, collects various examination results, standardises lung ultrasound recording standards, and mainly uses linear array probes for scanning (small convex array probes can be used for obese or elderly individuals with thicker chest walls). Under lung ultrasound, the child’s chest wall is divided into six areas: anterior, lateral, and posterior chest walls, on the ground of the sternal line, axillary front line, posterior axillary line, and spinal line. Each area is further divided into two zones, upper and lower, with transverse and longitudinal scans to record the ultrasound findings of each chest region. Abnormal ultrasound manifestations should be specific to the intercostal space to determine whether there are lung parenchymal lesions, the location, size, and number of consolidations, whether the pleural line is thickened, whether there is an increase or fusion of the B-line or B-line, whether there is pleural effusion, and whether there are abnormal ultrasound manifestations such as bronchial inflation sign, fluid filling sign, and pleural effusion. After an ultrasound examination, it records the pulmonary parenchymal lesions, determines the depth and quantity of pleural effusion, and collects ultrasound abnormal performance data. Then, this study classified and compared the areas where abnormal ultrasound findings were displayed on the children’s lungs. The presence of anomalies in each region is considered a positive feature, while those not detected are considered negative features. It calculates the number of cases of abnormal ultrasound manifestations and compares them.

### PCT and SAA inspection methods

The PCT and SAA levels recorded in the patient’s case were collected, and the data was on the ground of 3–4 ml of fasting elbow vein blood within 24 hours after admission, which was tested by the biochemical laboratory of our hospital’s laboratory. PCT is a serum sample (separated by centrifugation at 3000 r for 5 minutes), and SAA is a whole blood sample. They were tested using Yahuilong iFlash 3000 and a specific protein analyser. PCT was performed using the chemiluminescence method, and SAA was performed using the scattering turbidimetry method. Two personnel with years of experience in inspection operations strictly operate the instructions for Siemens’ relevant reagents and supporting instruments. The standard value of PCT is 0–0.5 μg/L, and the standard value of SAA is 1–10 mg/L.

### Statistics

Statistical software SPSS 22.0 was used to process the data, and Bartlett’s homogeneity of variance test and Shapiro Wilk’s normality test were used to measure the data. Both tests confirmed the homogeneity of variance and approximately followed a normal distribution, described by (x̄±s), and an independent sample t-test was used to compare the two groups. The number of use cases, or n (%), represents the counting data, and χ^2^ is used to test it. Using the MPP group as the positive sample and the non-MPP group as the negative sample, independent influencing indicators were determined using binary logistic regression analysis and combined with the Receiver operating characteristic curve (ROC) curve to assess the diagnostic value of ultrasound abnormal feature data, PCT, and SAA levels for MPP. Then, it compares the area under the curve, confidence interval, sensitivity, specificity, and related standards as observation indicators. The Youden Index was calculated as the sum of sensitivity and specificity minus 1 and was used to evaluate the overall diagnostic effectiveness of each test. A higher Youden Index value indicates better diagnostic performance [21]. We used the logistic regression prediction model to calculate the formula *Y=constant*+β_1x1_+β_2x2_+.......β_nxn_. Among them, Y is the variable of the joint diagnostic model, constant is a constant, x is the data of independent influencing factors, and β is the correlation regression coefficient of influencing factors. It uses GraphPad8.0.2 software to draw a ROC curve comparison chart. Correction level α=0.05.

## Results

A total of 80 children with community-acquired pneumonia were divided into two groups: 26 with Mycoplasma pneumoniae pneumonia (MPP) and 54 without MPP. The two groups showed no significant differences in gender, age, clinical manifestations, course of disease, and history of antibiotic use. Specifically, the MPP group had 18 males and 8 females, with a mean age of 7.62±1.50 years, while the non-MPP group had 32 males and 22 females, with a mean age of 6.87±2.33 years. The two groups also showed no significant differences in clinical manifestations such as fever, cough, and panting (P>0.05). The disease course was also similar between the two groups, with a mean of 7.19±3.12 days in the MPP group and 6.65±3.23 days in the non-MPP group. Additionally, there was no significant difference in the history of antibiotic application between the two groups, with 7 cases in the MPP group and 12 cases in the non-MPP group (P>0.05). However, significant differences were observed between the two groups in three ultrasound findings: B-line increase/fusion (P=0.024), a small amount of pleural effusion (P=0.046), and bronchogram (P=0.040). The MPP group had 14 cases of consolidation with a diameter of less than 1 cm, 6 cases with a diameter of 1–3 cm, and 12 cases with a diameter greater than 3cm. In contrast, the non-MPP group had 18 cases of consolidation with a diameter of less than 1 cm, 14 cases with a diameter of 1–3 cm, and 21 cases with a diameter greater than 3 cm.

The MPP group had 12 cases of consolidation with 1–3 pieces, while the non-MPP group had 21 cases with 1–3 pieces and 11 cases with a diameter greater than 3 cm.

**Figure 1:**
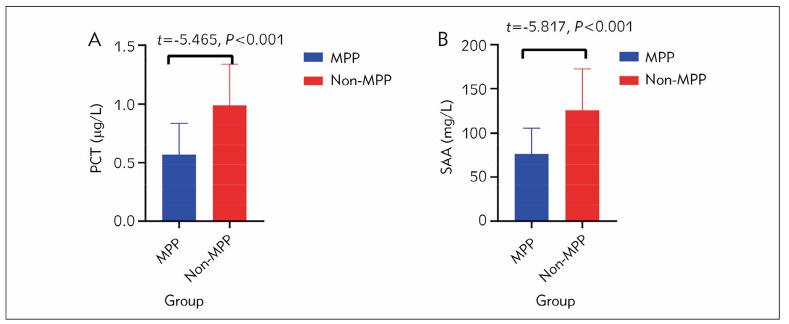
Comparison of plasma PCT and SAA examination results between two groups of patients Note: A represents the comparison of PCT levels between the two groups. B is the comparison of SAA levels between the two groups.

Figure 1 shows that the average level of PCT in the MPP group was 0.57±0.27 μg/L, significantly lower than the 0.99±0.35 μg/L in the non-MPP group. The difference in PCT between the two groups was statistically significant (*P*<0.001). The average level of SAA in the MPP group was 76.52±29.26 mg/L, significantly lower than the 126.12±46.34 mg/L in the non-MPP group. The difference in SAA between the two groups was also statistically significant (*P*<0.001).

Table 2 shows that the increase/fusion of B-line, PCT, and SAA are protective factors for MPP, with OR=0.091, 0.005, 0.941, and 95% CI of 0.012~0.696, 0.000~0.157, and 0.910~0.972, respectively. A small amount of pleural effusion is a risk factor for MPP, with an OR of 14.803 and a 95% CI of 1.775–123.466. The above four variables reached statistical significance in the model (*P*<0.05). According to the size of Wald values, SAA has the highest and most significant impact on the model. The Wald value of B-line increase/fusion is the smallest, which has the smallest impact on the model. The model is well established and has certain predictive value for MPP.

**Table 2:** Comparison of logistic regression analysis model results.

Variable	B	SE	Wald χ^2^	* P *	* OR *	* 95%CI *
B line increase/fusion	-2.402	1.040	5.331	0.021	0.091	0.012~0.696
Small amount of pleural effusion	2.695	1.082	6.200	0.013	14.803	1.775~123.466
PCT	-5.362	1.789	8.977	0.003	0.005	0.000~0.157
SAA	-0.061	0.017	13.351	0.000	0.941	0.910~0.972
constant	9.199	2.482	13.733	0.000	9887.740	

**Figure 2:**
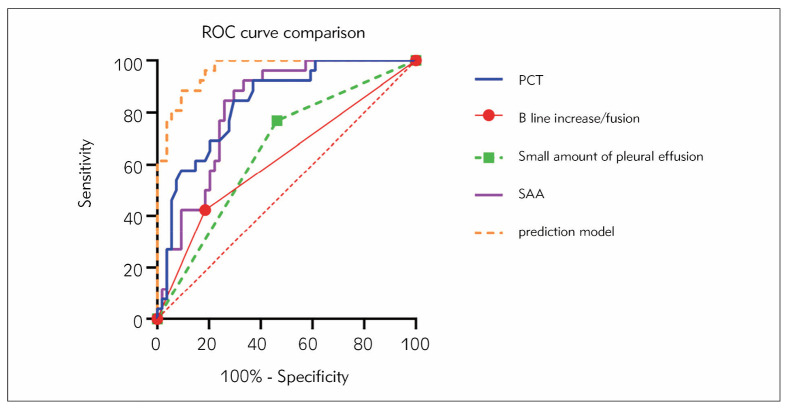
Comparison of ROC curves.

Constructing a logistic regression prediction model with MPP occurrence as the dependent variable=9.199–2.402 * B-line increase/fusion+2.695 * small amount of pleural effusion -5.362 * PCT- 0.061 * SAA. The sensitivity of the prediction model is lower than that of PCT and SAA, but the specificity is higher, with a Jordan index of 0.792 and an ACU of 0.963. Based on the relevant standard of>-0.471, the diagnostic efficacy comparison results are shown in Table 3, and the ROC curve comparison chart is shown in Figure 2. The diagnostic tests can be ranked in order of superiority based on the Youden Index as follows: prediction model (0.792), SAA (0.590), PCT (0.553), a small amount of pleural effusion (0.306), and B line increase/fusion (0.238).

**Table 3:** Comparison of Diagnostic Effectiveness.

variable	Sensitivity	Specificity	AUC	Youden index	Relevant standards
B line increase/fusion	42.31	81.48	0.619	0.238	No relevant images
Small amount of pleural<br>effusion	76.92	53.70	0.653	0.306	Presence of relevant<br>images
PCT	92.31	62.96	0.835	0.553	0.85 ng/mL
SAA	92.31	66.67	0.821	0.590	110.4 mg/L
prediction model	88.46	90.74	0.963	0.792	-0.471

## Discussion

This study evaluates the application value of lung ultrasound combined with plasma PCT and SAA in diagnosing mycoplasma pneumonia in children. The research results show that compared with non-MPP, MPP children showed significant differences in lung ultrasound imaging, including B-line increase/fusion, small pleural effusion, PCT, and SAA levels. Individuals with features such as increased/fused B-line, elevated PCT, and elevated SAA have a lower risk of developing MPP, while a small amount of pleural effusion is a risk for MPP. Wen et al. [22] show that MPP in children is a localised lesion with a lighter inflammatory response than bacterial pneumonia. Zheng et al. [23] also show a slight increase in inflammatory markers of MPP in children compared to bacterial pneumonia. The study by Fan et al. [24] also shows that there was no significant increase in PCT and CRP levels in children with mycoplasma pneumonia. Meanwhile, studies [25] [26] show that MPP has characteristics of interstitial changes in the lung, with manifestations such as widening of the pulmonary hilum and pleural fluid exudation. The research demonstrates that only a few cases are accompanied by pulmonary solid degeneration and necrosis [27]. The hilar region is the location of the lung’s serous channels. When inflammatory infiltration or fibrosis occurs, it can cause significant interstitial changes in the hilar region. An increase in B-line/fusion accompanies the widening of the hilar region. The exudation of pleural fluid can lead to a small amount of pleural effusion in children. Therefore, LUS can be used to differentiate MPP from other pneumonia pathogens.

This study combines lung ultrasound, PCT, and SAA to construct a logistic regression prediction model. It was found that the logistic regression model established based on the above four variables has a good predictive value for MPP. This can further improve the diagnostic accuracy of MPP in children.

The research demonstrates that mycoplasma ranks second in bacterial infections [28]. Mycoplasma itself is less invasive and does not release many inflammatory factors like bacteria, viruses, and other pathogens, producing many acute proteins in liver cells [29]. Meanwhile, children’s immune function is immature, and their response to pathogens such as mycoplasma is relatively slow, resulting in relatively less production of inflammatory factors.

Compared with other studies, this study, such as Wang et al. [15], used CT radiomics technology to distinguish between mycoplasma pneumonia and streptococcal pneumonia in children. Due to the lack of practical imaging diagnostic tools, early diagnosis of refractory Mycoplasma pneumoniae pneumonia (RMPP) is challenging. LUS is an emerging tool for diagnosing pneumonia in children [30]. LUS can evaluate the effectiveness of bronchial lavage through pulmonary complications and atelectasis, providing a reference for the clinical treatment of MPP [30]. This information suggests that lung ultrasound imaging indicators can provide certain pathogenic information. Meanwhile, Yang et al. [31] report on mycoplasma pneumonia in children with pulmonary embolism, indicating pulmonary interstitial changes. The B-line results of this study are consistent with them. Zhang et al. [32] report the CT manifestations of viral pneumonia, indicating differences in imaging characteristics among different pathogens. These studies all reflect the differential value of lung ultrasound in MPP to some extent.

Meanwhile, Jiang et al. [20] show that although SAA was elevated, it was impossible to differentiate MPP in children from other pneumonia alone. The research demonstrates that dynamic monitoring of serum PCT and CRP indicators can evaluate changes in the condition [33] [34]. Chen et al. found that the level of PCT is related to the severity of Mycoplasma pneumonia, with higher levels of PCT leading to higher levels of MPP infection [35]. There is no direct explanation in the literature regarding the combined detection of lung ultrasound. However, considering that mycoplasma pneumonia often manifests as interstitial lung lesions, lung ultrasound examination can reveal imaging changes of mycoplasma pneumonia. Future research will further expand the sample size and combine multicenter studies to accurately set the optimal diagnostic cutoff value to improve the accuracy of diagnostic tests. This study also has certain shortcomings, such as not observing the treatment and prognosis of the patients. On the grounds of MPP, glucocorticoids [34], azithromycin, and even minocycline [35] can be used for treatment. Mean while, this study cannot rule out MPP infection accompanied by lung infections caused by other pathogens, such as Karakayalı’s report of cases of coinfection with Mycoplasma and Staphylococcus aureus [36]. In the future, further research should be conducted on the early identification of MPP accompanied by other pathogen infections or refractory MPP infections.

The innovation of this study lies in the first combination of lung ultrasound and serum inflammatory markers PCT and SAA to improve the diagnostic accuracy of mycoplasma pneumonia in children. The results of this study have important guiding significance for optimising the diagnosis plan of pediatric pneumonia and can promote the transformation of the clinical diagnosis process of pediatric pneumonia. This enables differential diagnosis of MPP to no longer rely on a single pathogenic test but evolve towards a comprehensive approach that integrates multiple test results. In the future, it is necessary to expand the sample size further to verify the accuracy of the diagnostic results of this study and further improve the diagnostic efficiency of children with community-acquired pneumonia, especially myco plasma pneumonia. This can shorten the diagnosis time, reduce the misdiagnosis rate, and promote the progress of diagnostic technology for pediatric pneumonia. Also, this study has several limitations, including a small sample size of 80 children, a short study period of one year, and a single-centre design, which may not represent the larger population and may not capture the variability of Mycoplasma pneumoniae cases throughout the year. The study also lacks a control group of healthy children, which would have provided a baseline for comparison. The study also did not normalise PCT and SAA levels to account for individual variability, and the study did not include long-term follow-up to assess the predictive value of the biomarkers and lung ultrasound for disease progression and outcome. The study also did not compare the diagnostic value of PCT and SAA with other biomarkers, such as CRP and LDH, commonly used in diagnosing MPP

## Conclusion

The diagnostic value of LUS in pediatric mycoplasma pneumonia should be given more attention. LUS shows long B-line opacity and pulmonary consolidation, common imaging features of Myco plasma pneumonia. In some cases, a small amount of pleural effusion can be seen. These characteristics suggest a higher likelihood of Mycoplasma infection and pulmonary interstitial lesions. Meanwhile, a significant increase in PCT and SAA levels is also an important laboratory manifestation of community-acquired pneumonia caused by mycoplasma. By comprehensively applying different examination methods, the accuracy of distinguishing MPP from non-MPP pneumonia can be improved. For example, lung ultrasound results and biochemical marker detection should be combined to facilitate early differential diagnosis in the diagnosis process of cases with atypical clinical manifestations. Delayed diagnosis of MPP will affect disease control and drug treatment options. In addition, dynamically observing the changes in these indicators can also monitor the progress of the disease and evaluate the treatment effect. For example, the decrease in PCT levels and improvement in symptoms indicate the effectiveness of anti-infection treatment. On the contrary, if the examination results do not match clinical symptoms, an evaluation of the diagnosis is necessary.

## Dodatak

### Funding

The research is supported by the Jilin Province Health Science and Technology Capacity Enhance ment Project, The application of Lung Ultrasound in diagnosing and following up Pneumonia in Children (No. 2022LC037).

### Conflict of interest statement

All the authors declare that they have no conflict of interest in this work.
